# *Enterobius vermicularis* infection: prevalence and risk factors among preschool children in kindergarten in the capital area, Republic of the Marshall Islands

**DOI:** 10.1186/s12879-019-4159-0

**Published:** 2019-06-18

**Authors:** Chia-Kwung Fan, Ting-Wu Chuang, Ying-Chieh Huang, Ai-Wen Yin, Chia-Mei Chou, Yu-Ting Hsu, Ramson Kios, Shao-Lun Hsu, Ying-Ting Wang, Mai-Szu Wu, Jia-Wei Lin, Kennar Briand, Chia-Ying Tu

**Affiliations:** 1 Department of Molecular Parasitology and Tropical Diseases, School of Medicine, College of Medicine, Taipei Medical University, Taipei, 110 Taiwan; 2 Graduate Institute of Medical Sciences, College of Medicine, Taipei Medical University, Taipei, 110 Taiwan; 3 Division of Tropical Medicine, International Master/PhD Program in Medicine, College of Medicine, Taipei Medical University, Taipei, 110 Taiwan; 4 Master Program in Global Health and Development, College of Public Health, Taipei Medical University, Taipei, 110 Taiwan; 5 Taiwan Health Center in Majuro, Majuro, Republic of the Marshall Islands; 6 Department of Public Health, Majuro, Republic of the Marshall Islands; 7 Superintendent Office, Taipei Medical University-Shuang-Ho Hospital, New Taipei City, 235 Taiwan; 8 Department of International Medical Affairs, Taipei Medical University-Shuang-Ho Hospital, New Taipei City, 235 Taiwan; 9 Majuro Hospital, Ministry of Health, Majuro, Republic of the Marshall Islands

**Keywords:** *Enterobius vermicularis*, Preschool children, Majuro City, Republic of Marshall Islands

## Abstract

**Background:**

*Enterobius vermicularis* (pinworm) is one of the most common human parasitic helminths, and children are the most susceptible group. Some behavioral and environmental factors may facilitate pinworm infection. In the Republic of the Marshall Islands (RMI), the status of pinworm infections among children remains unknown.

**Methods:**

In Majuro City, there are 14 kindergartens with a total of 635 preschool children (PSC) whose age range of 5~6 years. The present investigation attempted to determine the pinworm prevalence and associated risk factors as well as investigate whether eggs contaminated the clothes of PSC or the ground and tables in classrooms of 14 kindergartens. Informed consent form and a self-administered questionnaire were given to parents prior to pinworm screening. Perianal specimens were collected by an adhesive scotch tape method, and clothing of belly and hip sites and the ground and tables of the classrooms were inspected using a cellophane tape method to detect any eggs contamination.

**Results:**

In total, 392 PSC (5.28 ± 0.56 yrs. old) participated in this project. The overall prevalence of pinworm infection was 22.4% (88/392). Boys (24.5%) had higher prevalence than girls (20.31%) (*p* = 0.32). PSC aged > 5 years (32.77%) showed a significantly higher prevalence than those aged ≤5 years (17.95%) (*p* = 0.01). A univariate analysis indicated that PSC who lived in urban areas (22.95%) had a higher prevalence than those who lived in rural areas (20.69%) (*p* = 0.69). The employment status of the parents showed no association with the pinworm infection rate (*p* > 0.05). A logistic regression analysis indicated that “having an older sister” produced a higher risk of acquiring pinworm infection for PSC compared to those who did not have an older sister (OR = 2.02; 95%CI = 1.05~3.88; *p* = 0.04). No significant association between various other risk factors and pinworm infection was found (*p* > 0.05). Also, no eggs contamination was found on the clothes of the belly and hip sites or on the ground and tables in the 14 kindergartens.

**Conclusions:**

Mass screening and treatment of infected PSC are important measures in pinworm control in the RMI.

**Electronic supplementary material:**

The online version of this article (10.1186/s12879-019-4159-0) contains supplementary material, which is available to authorized users.

## Background

*Enterobius vermicularis* (pinworms) is one of the most common human parasitic helminths and by one estimation, about 200 million people worldwide are supposedly infected, with children aged 5~10 years old accounting for over 30% of cases [1].

Regardless of one’s particular socioeconomic level, race, or culture, pinworm infection can be facilitated by certain factors such as poor personal or group hygiene, and overcrowding in preschools, schools, orphanages, and family groupings [2, 3]. These conditions favor pinworm eggs transmission from person to person, directly via the anus-to-mouth route and finger contamination or indirectly by contaminated objects, e.g., toys, classroom tables, chairs, or the ground [2, 4]. Since personal hygiene and exposure are important transmission factors, preschool-aged children (PSC) who live in crowded environments such as kindergartens are the most common group susceptible to pinworm infection [2].

Adult males measure 2 to 5 mm, and the females measure 8 to 13 mm. The cecum of the large intestine is the major site for pinworms to live and the gravid female migrates at night to lay up to 15,000 eggs. Ingested eggs hatch in the duodenum, and larvae mature during their migration to the large intestine. In the absence of host autoinfection, infestation usually lasts only four to six weeks [1, 2]. In general, female worms release their eggs on the skin near the anus, and some eggs may detach from the perianal region and lodge on clothing, bedding, and other surfaces such as the ground or tables and chairs [2]; therefore, children may acquire an infection through ingestion of eggs-contaminated foods or inhalation of infective eggs in the dust or retrograde migration of hatched larvae from the anus to the intestines. This infection is more common in temperate than in tropical districts, although recent studies indicated that a prevalence of over 20% is not uncommon in many parts of the world [1, 3, 4].

Although pinworm infection may be symptomless in most patients, some of them may suffer perianal pruritus, insomnia, restlessness, and irritability, particularly children [1]. It should be stressed that pinworms may cause serious morbidity such as appendicitis and eosinophilic enterocolitis, and sometimes ectopic infections can result in pelvic inflammatory disease or urinary tract infections in females [2, 5, 6].

The appropriate diagnostic choice is to employ a cellophane tape test or scotch tape method for screening instead of stool examinations since eggs can be detected in only about 5% of fecal samples; in other words, the prevalence of pinworm infection is generally underestimated due to the difficulty of detecting pinworm eggs by stool examinations [7, 8]. Although effective treatment has been established for decades, the control of pinworm infection remains a challenge due to reinfection, incomplete treatment, and its characteristic of easy transmission [8].

In the Republic of the Marshall Islands (RMI), the status of pinworm infection among children is supposedly high; nevertheless, until recently its exact prevalence has remained unknown. The country is situated in the Pacific Ocean, with a climate of high temperatures and moisture. The present investigation attempted to determine the prevalence of pinworm infection and associated risk factors by questionnaire interviews with the help of public health nurses. We also investigated whether eggs contaminated participants’ clothing or the ground and tables in kindergarten classrooms. This information will help establish baseline data for the Marshallese Ministry of Health (MOH) to enact effective measures against pinworm infection and transmission.

## Methods

### Geography of the Republic of the Marshall Islands and Majuro City

**Fig. 1:**
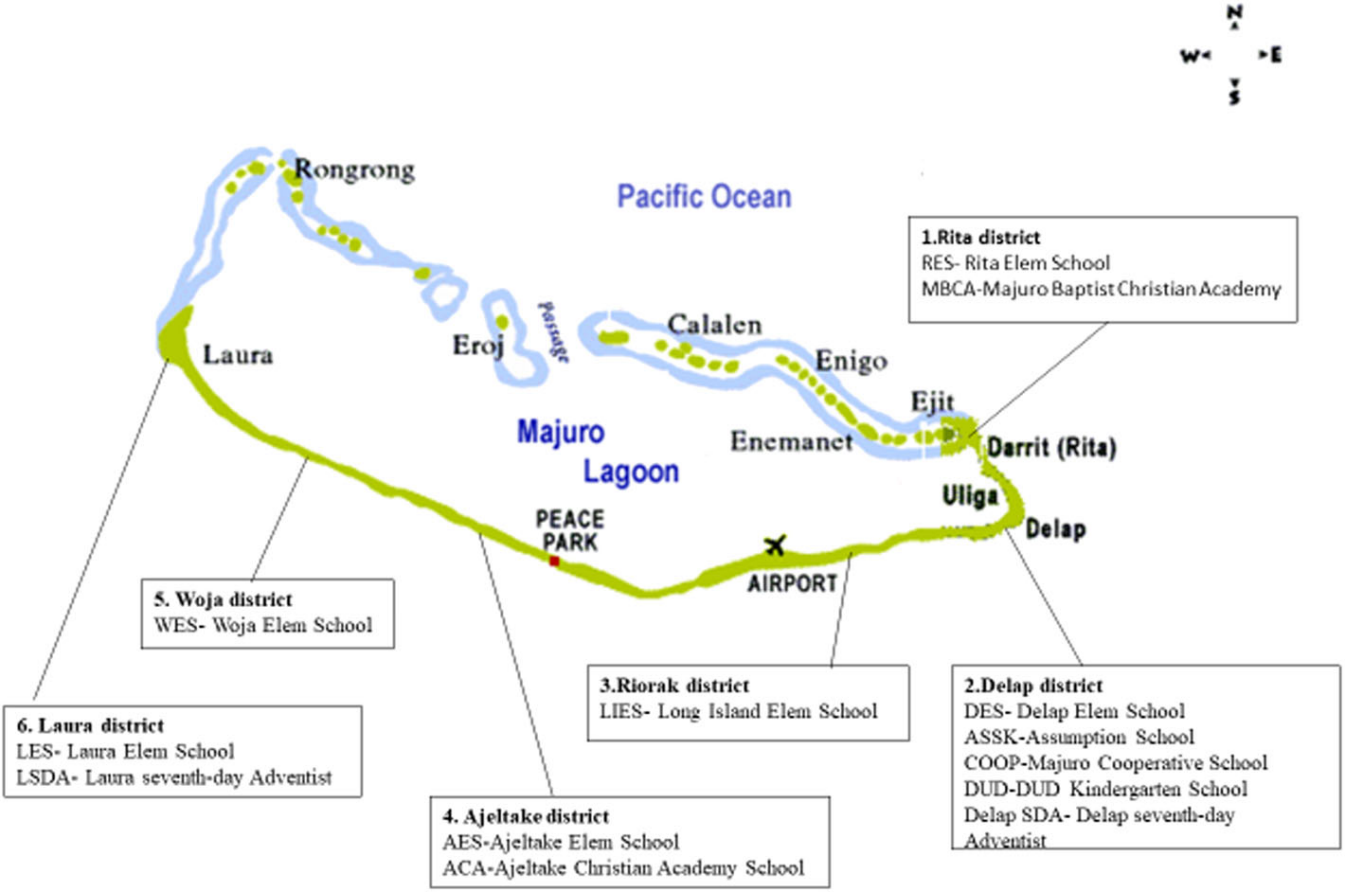
Map indicated the location of participant kindergartens at Majuro City, Republic of the Marshall Islands. Map is cited from the website: https://scottislandgrant.weebly.com/live.html

The RMI is an island nation situated in the central Pacific Ocean between 4° and 14°N latitude and 160° and 173°E longitude. Majuro Atoll, a large coral atoll of 64 islands, is a legislative district of the Ratak Chain of the RMI. Majuro Atoll has a land area of 3.7 mi^2^ and encloses a lagoon of 295 km^2^. The RMI has a total population of 52,560. The primary population center, named Majuro, is the capital and largest city in the RMI. Its characteristic climate is tropical, and the economy primarily relies on agriculture, fishery, and support from the United States. The major ethnic group is Micronesian [9]. This study was conducted in 14 kindergartens of six administrative areas in Majuro City according to suggestions by the MOH, RMI (Fig. 1).

### Study population and subject selection

This study was conducted from October 1 to 29, 2017. In Majuro City, there are 14 kindergartens in six administrative areas with a total of 635 PSC with an age range of 5~6 years. Prior to commencing the pinworm screening campaign, several explanatory meetings concerning the purpose of this screening project were held for parents and guardians. We also reminded them not to bathe their children on the morning of the collection day to avoid possible false-negative findings, and we distributed an informed consent form and a self-administered questionnaire to them. In addition to PSC, surfaces of tables and the ground in classroom were also examined for pinworm eggs contamination. Since no pilot study had previously been conducted for pinworm screening in the RMI, the sample size was determined using the general formula, *n* = z^2^p (1 − p)/d^2^ where, *n* is the sample size, z (1.96) is the standard deviation at a 95% confidence interval (CI), p is the prevalence (23%) based on the infection rate of intestinal parasites in the RMI from a previous study [10], and d is the allowed relative error (0.05) [11]. The minimum sample size from the calculation was 273 PSC.

### Parasitological survey by employing the adhesive scotch tape and cellophane tape methods

In each participating child, a one specimen from the perianal site was collected by the adhesive scotch tape method, and two specimens including one from the belly and one from hip site of the clothing were collected by the cellophane tape method [1, 4]. In the meantime, the ground in the classroom was divided into nine collection sites (upper, middle, and lower parts of the left, middle, and right portions), and nine collections from each ground and 3~6 tables located on the ground in each kindergarten were also randomly inspected once per table using the cellophane tape method to detect any eggs contamination [4, 12].All of the specimen collected from kids, grounds and tables from each kindergarten were kept in the storage boxes and transported to medical laboratory of Majuro hospital immediately under room temperature and one qualified technician examined all of the specimen.

### Questionnaire survey to determine risk factors of pinworm infection

A questionnaire was given to the parents or guardians of each child, asking about the child’s gender, the parents’ occupation, and family members. In addition, information concerning risk factors, including washing the hands before eating, washing the hands after using toilet facilities, finger sucking, fingernail trimming, the way and frequency of bathing, the type of floor, and the frequency of cleaning the bedding, was also obtained from participants [12].

### Chemotherapy and follow-up

Infected children and their families were administered with a single dose of albendazole (200 mg/tablet) by public health nurses, MOH, RMI. A follow-up examination was conducted 2 weeks after chemotherapy by a medical technician in Laboratory Medicine, Majuro Hospital, RMI.

### Statistical analysis

Differences in the prevalence of infection based on independent variables including gender, age group, residence, and parents’ employment status were determined by a Chi-squared (*x*^2^) test. The univariate crude odds ratio (OR) and 95% confidence interval (CI) were used to determine associations between independent variables, risk factors, and infection, and *p* values of < 0.05 were considered statistically significant. All statistical analyses of data from the questionnaire and parasitological examination were conducted using SAS vers. 9.3 software (SAS Institute, Cary, NC, USA).

### Ethics approval and consent to participate

The research protocols were approved by the Institutional Review Board of Shuang Ho Hospital, Taipei Medical University (TMU-JIRB no. N201708019), and they were also approved by the MOH, RMI. Informed consent form was obtained from participant parents/guardians to allow their kids to participate in this project. Meanwhile, participants were informed whether all of their data were permitted to consent for publication was also obtained in the informed consent.

## Results

In Majuro City, there are 14 kindergartens in six administrative areas with a total of 635 PSC. In the present study, 392 PSC, with a mean age of 5.28 ± 0.56 years participated in this project for a participation rate of 61.7% (392/635). We found that 88 PSC had pinworm infection, giving an overall prevalence of 22.2% (88/392) (Table 1). Boys (24.5%, 49/200) had a slightly higher prevalence than girls (20.31%, 39/192) (*p* = 0.32) (Table 1). PSC aged > 5 years had a significantly higher prevalence (32.77%, 39/119) than that of PSC aged ≤5 years (17.95%, 49/273) (*p* = 0.01) (Table 1). A univariate analysis of the family background associated with pinworm infection indicated that PSC who lived in an urban area (Rita, Delap, or Riorak) (22.95%, 70/305) had a slightly higher prevalence than those who lived in a rural area (Ajeltake, Woja, or Laura) (20.69%, 18/87) (*p* = 0.69) (Table 1). Also, no significant relationship was found between the employment status of father or mother and the pinworm infection rate in children (*p* > 0.05) (Table 1). Interestingly, PSC who had an older sister (26.47%, 63/238) had a statistically higher prevalence than those who did not have an older sister (16.89%, 25/148) (*p* = 0.03) (Table 1). However, having an older or younger brother produced no significant difference in the prevalence of pinworm infection (*p* > 0.05) (Table 1). All demographic results are given in Table 1.

**Table 1:** Demographic characteristics of *Enterobius vermicularis* infection among preschool children from kindergartens in the capital area of the Republic of the Marshall Islands

Variable	*Enterobius vermicularis*		
	Positive cases	Infection rate (%)	*p* value^a^
Gender			
Female (*N* = 192)	39	20.31	0.32
Male (*N* = 200)	49	24.50	
Age			
≤ 5 years (*N* = 273)	49	17.95	0.001^a^
> 5 years (*N* = 119)	39	32.77	
Urban residence			
No (*N* = 87)	18	20.69	0.65
Yes (*N* = 305)	70	22.95	
Father employed			
No (*N* = 57)	12	21.05	0.69
Yes (*N* = 312)	73	23.40	
Mother employed			
No (*N* = 243)	62	25.51	0.08
Yes (*N* = 136)	24	17.65	
Have an older brother			
No (*N* = 148)	33	22.30	0.88
Yes (*N* = 240)	55	22.92	
Have an older sister			
No (*N* = 148)	25	16.89	0.03^a^
Yes (*N* = 238)	63	26.47	
Have a younger brother			
No (*N* = 197)	46	23.35	0.69
Yes (*N* = 189)	41	21.69	
Have a younger sister			
No (*N* = 200)	45	22.50	0.95
Yes (*N* = 189)	43	22.75	

^a^ By Chi-square test

The logistic regression analysis further revealed that only the factor of having an older sister showed a higher risk of acquiring pinworm infection for PSC compared those who did not have an older sister (OR = 2.02; 95% CI = 1.05~3.88; *p* = 0.04) (Table 2). However, from the logistic regression analysis, we found no significant association between various variables and pinworm infection (*p* > 0.05) (Table 3). Also, we found no eggs contamination on the clothing of the belly or hip sites of each PSC by examining 784 cellophane tape-collected specimens as well as 182 cellophane tape-collected specimens from the ground and tables in 14 classrooms of the 14 kindergartens (data not shown).

**Table 2:** Logistic regression analysis of *Enterobius vermicularis* infection among preschool children from kindergartens in the capital area of the Republic of the Marshall Islands

Variable	*Enterobius vermicularis*		
	OR	95% CI	*p* value
Gender			
Female	1.00		0.51
Male	1.21	0.69~2.09	
Age			
≤ 5 years	1.00		0.08
> 5 years	1.66	0.95~2.90	
Urban residence			
No	1.00		0.16
Yes	1.65	0.82~3.32	
Father employed			
No	1.00		0.33
Yes	1.43	0.64~3.19	
Mother employed			
No	1.00		0.23
Yes	0.69	0.38~1.26	
Have an older brother			
No	1.00		0.38
Yes	0.76	0.42~1.40	
Have an older sister			
No	1.00		0.04*
Yes	2.02	1.05~3.88	
Have a younger brother			
No	1.00		0.97
Yes	0.99	0.56~1.74	
Have a younger sister			
No	1.00		0.71
Yes	0.90	0.51~1.58	

*OR* odds ratio, *CI* confidence interval

**Table 3:** Logistic regression analysis of risk-factors for *Enterobius vermicularis* infection among preschool children from kindergartens in the capital area of the Republic of the Marshall Islands

Variable	*Enterobius vermicularis*		
	OR	95% CI	*p* value
Washing hands before eating			
Infrequent	1.00		0.26
Frequent	2.56	0.49~13.25	
Washing hands after using toilet facilities			
Infrequent	1.00		0.42
Frequent	0.53	0.11~2.49	
Finger sucking			
No	1.00		0.81
Yes	0.89	0.35~2.29	
Keeping the fingernails short			
No	1.00		0.64
Yes	0.88	0.51~1.52	
Way of bathing			
Showering	1.00		0.89
Bathing in a tub	1.05	0.52~2.15	
Taking a bath after getting up			
No	1.00		0.49
Yes	1.60	0.41~6.24	
Bathing with the help of family members			
No	1.00		0.55
Yes	0.78	0.35~1.74	
Type of floor			
Ground floor, single-family detached or townhouse	1.00		0.33
Apartment	0.59	0.20~1.73	
Type of bed			
Matting	1.00		0.83
Wood or spring mattress	1.07	0.60~1.91	
Change bedding < 2 weeks			
No	1.00		0.74
Yes	1.45	0.16~13.59	
Share a bedroom with family members			
No	1.00		0.14
Yes	0.59	0.29~1.19	
Share a bed with family members			
No	1.00		0.80
Yes	1.09	0.57~2.07	

*OR* odds ratio, *CI* confidence interval

## Discussion

In general, the infection caused by *E. vermicularis* is relatively innocuous. Nevertheless, eggs deposition may cause perineal, perianal, and even vaginal irritation, and infected persons may try to relieve the irritation of the constant itching, possibly leading to potentially debilitating sleep disturbance, impaired concentration, emotional instability, or enuresis [3]. Furthermore, these uncomfortable symptoms can result in weight loss, urinary tract infections, and even acute or chronic appendicitis which can lead to death without appropriate surgical treatment [5, 6]. Therefore, children who exhibit perianal pruritus and nocturnal restlessness should be suspected of having pinworm infection [1, 3].

A previous study indicated that behavioral changes are frequently observed in pinworm-infected children who feel shameful and inferior due to having ‘worms’ [13]. However, children’s discomfort is often overlooked by parents, despite pinworm infection possibly causing developmental and/or health problems. In particular, mothers can become distraught when they think their child has been infected by ‘worms’ [3]. Therefore, treating pinworm infection can improve the quality of the child’s life, and campaigns and control measures to prevent pinworm are positively recognized by the majority of parents [4, 8].

Although adult worms directly seen by the naked eye and microscopic detection of eggs from feces allow a confirmative diagnosis, this is impractical, because adult worms are uncommonly seen around the anal area or in the stool, and eggs are only found in the stool of 5% of infected persons [1, 3]; thus, the scotch-tape test can serve as a quick and sensitive way to clinch a diagnosis [14].

In the present study, the overall prevalence of pinworm infection was 22.2% (88/392) in Marshallese PSC, and this infection rate is higher than those reported in Seoul, South Korea (9.5%, 113/1191) [8], Taipei, Taiwan (0.21%, 94/44163) [12], and Amol County, Iran (7.1%, 33/462) [15]; however, it was lower than rates seen in Cordobe Province, Argentina, where the prevalence of *E. vermicularis* in PSC was 43.4% [16], northern Thailand (25%) [17], and Timisoara, Romania (42.8%) [18].

The univariate analysis in the present study showed that the prevalence of pinworm infection was significantly higher in children aged > 5 years than in younger children aged ≤5 years, which agrees with results of other studies [8, 19]. This can be explained by play activity programs for children > 5 years old slightly differ from those of younger children aged ≤5 years, as they play outside at the kindergarten instead of taking a nap. Therefore, they have more opportunities to play with dirt and have greater frequency of physical contact with their friends than do younger children, thus they have a higher risk of acquiring pinworm infection. Although no significant difference was found in prevalences by gender, the rate in boys was slightly higher. These findings were similar to those reported previously [20–22] and it may be explained by gender differences in children, as girls having better personal hygienic habits than boys, thus protecting them from pinworm infection.

Interestingly, we found that children with an older sister had a significantly higher prevalence. This finding was also similar to that reported in Taiwan [12], indicating that transmission of the infection might occur in the family through school-aged siblings, particularly if an older sister is tasked with caring for younger sisters and brothers. Thus, in the RMI, an older sister might play an important role in transmitting pinworm eggs to her younger siblings.

Substantial studies have indicated that parents’ occupation is an important indicator of the socioeconomic status of the children; however, in the present study, the parents having or not having a job was not related to the opportunity for pinworm infection in PSC, although the occupation of the parents was also reported to be a risk factor for infection [4, 7, 8, 23].

It was reported that inadequate personal hygiene might increase the risk of pinworm infection among children, and significant factors associated with pinworm infection include playing on the floor, nail biting, failing to wash hands before meals, and living in non-apartment dwellings [24]. However, personal hygiene factors, such as finger sucking, keeping the fingernails short, the frequency and way of bathing, etc., were not found to be significantly associated with pinworm infection for Marshallese PSC in the present study. Although sharing a bed/bedroom with family members and the floor type were previously reported to be important risk factors in pinworm transmission [25], in the present study, we did not find such an association with pinworm infection. It was reported that environmental factors are important in the transmission of pinworm [4, 12, 25]. In our study, we did not find pinworm eggs contamination on tables or the ground in the classrooms or on the belly and hip sites of clothing from the children from the 14 participating kindergartens. This indicates that tables, the ground, and the children’s clothing might not be important transmission source for Marshallese PSC.

## Conclusion

Taken together, mass screening should continue, and infected PSC and their family members should be treated, both of which are important measures for controlling pinworm infection in the RMI.

## Additional file

Additional file 1: Anonymous original data for statistical analysis. (XLSX 83 kb)

## Data Availability

All of the original data has been appended in Additional file 1.

## Abbreviations

CI: Confidence interval
MOH: Ministry of Health
OR: Odds ratio
PSC: Preschool-aged children
RMI: Republic of the Marshall Islands
*x*^2^: Chi-squared test
